# Inhibition of Histone Deacetylation and DNA Methylation Improves Gene Expression Mediated by the Adeno-Associated Virus/Phage in Cancer Cells

**DOI:** 10.3390/v5102561

**Published:** 2013-10-22

**Authors:** Azadeh Kia, Teerapong Yata, Nabil Hajji, Amin Hajitou

**Affiliations:** 1 Phage Therapy Group, Department of Medicine, Imperial College London, Hammersmith Hospital Campus, London W12 0NN, UK; E-Mails: a.kia@ucl.ac.uk (A.K.); teerapong.yata09@imperial.ac.uk (T.Y.); 2 Epigenetic Group, Department of Medicine, Imperial College London, Hammersmith Hospital Campus, London W12 0NN, UK; E-Mail: n.hajji@imperial.ac.uk

**Keywords:** bacteriophage, phage-targeted gene transfer, cancer gene therapy, AAV/phage, Grp78, histone acetylation/deacetylation, DNA methylation

## Abstract

Bacteriophage (phage), viruses that infect bacteria only, have become promising vectors for targeted systemic delivery of genes to cancer, although, with poor efficiency. We previously designed an improved phage vector by incorporating cis genetic elements of adeno-associated virus (AAV). This novel AAV/phage hybrid (AAVP) specifically targeted systemic delivery of therapeutic genes into tumors. To advance the AAVP vector, we recently introduced the stress-inducible *Grp78* tumor specific promoter and found that this dual tumor-targeted AAVP provides persistent gene expression, over time, in cancer cells compared to silenced gene expression from the *CMV* promoter in the parental AAVP. Herein, we investigated the effect of histone deacetylation and DNA methylation on AAVP-mediated gene expression in cancer cells and explored the effect of cell confluence state on AAVP gene expression efficacy. Using a combination of AAVP expressing the GFP reporter gene, flow cytometry, inhibitors of histone deacetylation, and DNA methylation, we have demonstrated that histone deacetylation and DNA methylation are associated with silencing of gene expression from the CMV promoter in the parental AAVP. Importantly, inhibitors of histone deacetylases boost gene expression in cancer cells from the *Grp78* promoter in the dual tumor-targeted AAVP. However, cell confluence had no effect on AAVP-guided gene expression. Our findings prove that combination of histone deacetylase inhibitor drugs with the *Grp78* promoter is an effective approach to improve AAVP-mediated gene expression in cancer cells and should be considered for AAVP-based clinical cancer gene therapy.

## 1. Introduction

Targeting therapeutic genes efficiently and specifically to tumors following systemic administration would present a major advance in cancer gene therapy. Animal viruses provide superior gene delivery vectors; however, they have had limited success in targeted systemic cancer gene therapy because of uptake by the liver and reticulo-endothelial system, broad tropism for normal tissues, and neutralizing antibodies [1]. Alternatively bacteriophage (phage), bacterial viruses, have attracted attention as safe vectors for targeted systemic gene delivery. Bacteriophage infect bacteria only and have no intrinsic tropism for mammalian or plant cells. However, if a mammalian ligand is displayed on the phage surface (targeted phage), the ligand can bind to its specific receptor on eukaryotic cells resulting in entry and internalization of the bacteriophage into the cells, eventually leading to the delivery of a eukaryotic transgene cassette inserted within the phage genome [2]. Despite some attractive features, bacteriophage viruses are still considered poor vectors. Unlike eukaryotic viruses, they have no intrinsic strategies for delivering genes to eukaryotic cells; however, we have previously reported that phage gene transfer efficacy can be improved [3,4,5]. We designed an improved phage vector, which incorporated a mammalian transgene cassette flanked by inverted terminal repeats (ITRs) from adeno-associated virus (AAV2) into the bacteriophage genome [3,6,7,8]. In this vector, named AAV/phage or AAVP, the targeted M13-derived phage served as a carrier to deliver the AAV2 transgene cassette. Tumor targeting was achieved by displaying the RGD4C (CDCRGDCFC) ligand, on the phage capsid, to target overexpressed alpha v integrin receptors in tumors [3,9,10]. This phage vector showed improved gene transfer efficiency, which was associated with better fate of the vector genome through maintenance of the entire mammalian transgene cassette, better persistence of extrachromosomal vector DNA, and formation of concatamers of the AAV transgene cassette, or a combination of these non-mutually exclusive mechanisms [3,7]. Transgene expression by RGD4C/AAVP was driven by a cytomegalovirus (*CMV*) promoter, largely utilized in many gene therapy vectors to drive strong and constitutive expression of transgenes. However, this promoter is not specific and undergoes silencing by mammalian host cells [11,12,13]. Thus, to further improve AAVP at the genome level, we introduced a eukaryotic tumor specific promoter of the glucose regulated protein 78 (*Grp78*) to drive gene expression [14]. The *Grp78* gene is selectively induced in tumors, but its activity is not detectable in major normal tissues [15]. We therefore generated a dual tumor-targeted RGD4C/AAVP-*Grp78* vector containing the RGD4C tumor homing ligand and *Grp78* promoter [14]. In our previously published work, we reported that the double-targeted RGD4C/AAVP-*Grp78* provides persistent transgene expression over RGD4C/AAVP*-CMV* carrying the *CMV* promoter [14]. Our recent work reporting silencing of the *CMV* promoter in both U87 and 9L cancer cells is consistent with other studies [12,13,16]. Herein, we aimed to gain further insight into gene expression silencing from the RGD4C/AAVP*-CMV* phage vector, its persistence from RGD4C/AAVP-*Grp78* and subsequently improved AAVP-mediated gene expression in cancer cells. 

## 2. Results and Discussion

**Figure 1:**
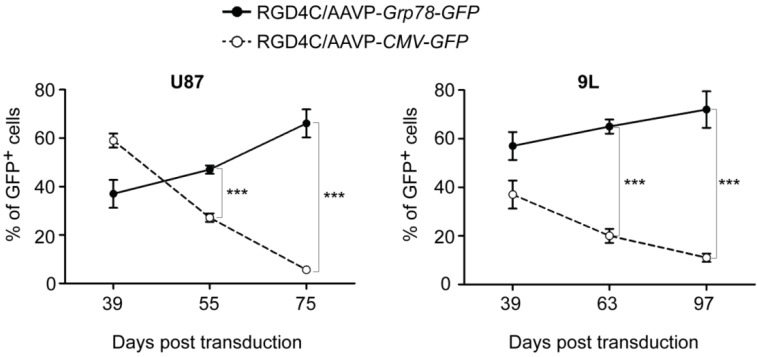
Persistence of gene expression from RGD4C/AAVP-*Grp78* and silencing of RGD4C/AAVP-*CMV*-mediated gene delivery in cancer cells. U87 and 9L glioblastoma cells were stably transduced with RGD4C/AAVP-*CMV-GFP* or RGD4C/AAVP-*Grp78-GFP* vectors. Then GFP positive cells were monitored by flow cytometry over a period of 39 to 75 days post-transduction of U87 cells, and 39 to 97 days post-transduction of 9L cells. This experiment was repeated three times with similar results, shown are data of one experiment. Statistical analyses were performed by using GraphPad Prism software (version 5.0). Error bars represent standard error of the mean (s.e.m). *p*-values were generated by ANOVA and denoted as follows: * *p* < 0.05, ** *p* < 0.01 and *** *p* < 0.001.

We monitored gene expression by AAVP in the human U87 and rat 9L glioblastoma cells over an extended time course by generating stably transduced cells with vectors carrying *puro^R^* gene that confers puromycin resistance. A marked decrease in gene expression from the RGD4C/AAVP*-CMV* phage vector was observed over time in U87 and 9L cells; in contrast, no silencing of *Grp78*-regulated gene expression was detected following cell transduction with the double-targeted RGD4C/AAVP-*Grp78* phage (Figure 1). 

**Figure 2:**
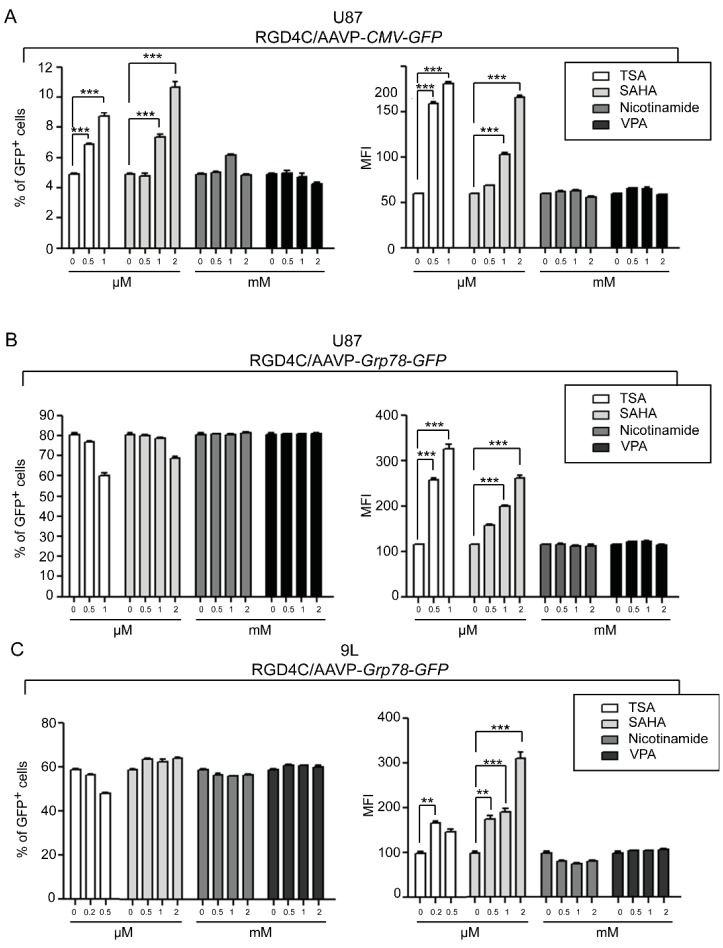
HDAC inhibitors restore gene expression from RGD4C/AAVP-*CMV* and boost RGD4C/AAVP-*Grp78*-mediated gene expression in cancer cells. U87 cells at day 75 post-transduction with (**A**) RGD4C/AAVP-*CMV-GFP* or (**B**) RGD4C/AAVP-*Grp78-GFP* were plated in 6 well plates, then treated 24 h later with various concentrations of the HDAC inhibitors TSA, SAHA, nicotinamide or VPA for three days; (**C**) 9L cells stably transduced with RGD4C/AAVP-*Grp78-GFP* were treated with various concentrations of the HDAC inhibitors at day 97 post-transduction as described above. Finally, U87 and 9L cells were collected and GFP positive cells as well as the MFI were analyzed by flow cytometry. The experiments were performed in triplicate, repeated three times with similar results, and a representative experiment is shown. Error bars represent standard error of the mean (s.e.m). *p*-values were generated by ANOVA and denoted as follows: * *p* < 0.05, ** *p* < 0.01 and *** *p* < 0.001.

**Figure 3:**
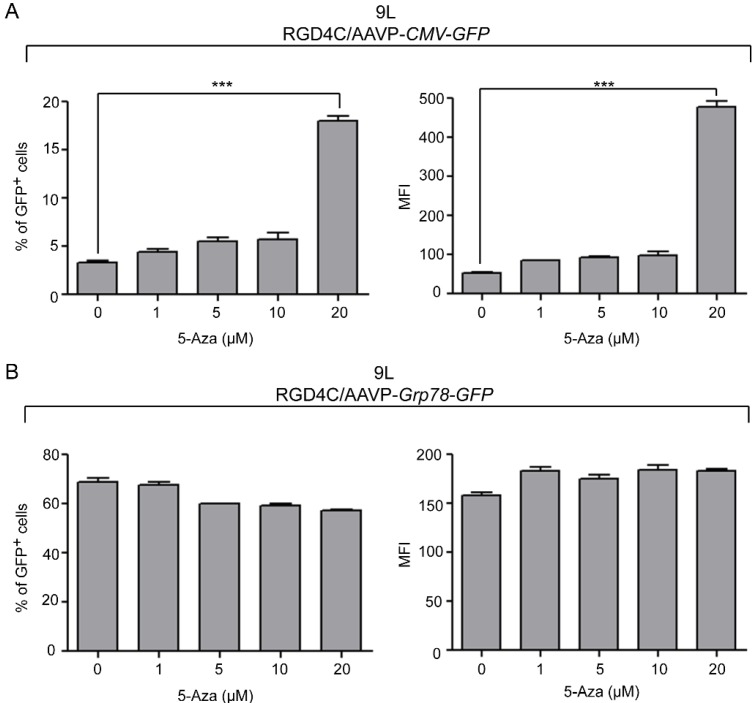
Treatment with the DNA methylation inhibitor 5-Aza restores gene expression from RGD4C/AAVP-*CMV* in 9L glioblastoma cells. 9L cells stably transduced with (**A**) RGD4C/AAVP-*CMV*-*GFP* or (**B**) RGD4C/AAVP-*Grp78*-*GFP* were kept for 97 days in culture, then plated in 6 well plates and treated 24 h later with increasing concentrations of 5-Aza. At day three, post 5-Aza treatment, cells were collected and GFP positive cells as well as the MFI were analyzed by flow cytometry. The experiments were repeated three times, in triplicate, with similar results and shown are data from a representative experiment. Statistical analyses were performed as described in Figure 1 and Figure 2.

Although the exact mechanisms of viral promoter silencing have remained mainly unknown, several studies have demonstrated the association of DNA methylation and histone deacetylation with inactivation of the *CMV* promoter [11,13,16,17]. Generally, both DNA methylation and histone acetylation statuses play major roles in the regulation of gene expression by providing transcription factors’ accessibility to gene promoters. The precise balance of acetylated and deacetylated states of histones is an important feature of gene regulation and the imbalance is found in many human cancers, often resulting from alterations in histone acetyltransferase (HATs) and histone deacetylase (HDACs) enzyme activities. Here, we quantified AAVP-mediated gene expression in the presence of HDAC inhibitors by using vectors expressing the green fluorescent protein (*GFP*), RGD4C/AAVP-*CMV-GFP* and RGD4C/AAVP-*Grp78-GFP*, and carrying the *puro^R^* to generate stable gene expression by stably transduced cells. Flow cytometry was used and both percentage of GFP positive cells and mean fluorescent intensity (MFI) were calculated by normalizing the results to parental non-transduced cells. As an initial experiment, we evaluated GFP expression in the human U87 cancer cells transduced with RGD4C/AAVP-*CMV-GFP* or RGD4C/AAVP-*Grp78-GFP* upon treatment with increasing concentrations of trichostatin-A (TSA), a pan-HDAC inhibitor. TSA is the first characterized organic HDAC inhibitor [18] widely utilized to study the reactivation of silenced viral constructs. In RGD4C/AAVP-*CMV-GFP*-transduced U87 cells, treatment with 0.5 µM and 1 µM TSA resulted in a significant increase of both percentage of GFP positive cells by 1.4- and 1.8-fold, and MFI by 2.7- and 3.0-fold, respectively (Figure 2A). These findings are consistent with previous reports demonstrating reactivation of the *CMV* promoter by TSA in U87 cells and other cell lines [12,19]. Interestingly, GFP expression in U87 cells stably transduced by RGD4C/AAVP-*Grp78-GFP* increased at the level of MFI only, upon TSA treatment, with no effect on GFP positive cells (Figure 2B). Next, we investigated additional HDAC inhibitors such as suberoylanilide hydroxamic acid (SAHA), which is structurally similar to TSA, as well as nicotinamide and valporic acid (VPA). SAHA treatment with 0.5 µM and 1 µM yielded results comparable to TSA and resulted in a dose dependent reactivation of gene expression in U87 cells transduced with RGD4C/AAVP-*CMV-GFP*, enhancing the percentage of GFP positive cells by 1.6- and 2.2-fold, as well as MFI by 1.8- and 2.8-fold, respectively (Figure 2A). Interestingly, in RGD4C/AAVP-*Grp78-GFP*-transduced cells and similar to TSA treatment, only the MFI was increased upon SAHA treatment while the percentage of GFP positive cells remained intact (Figure 2B). Finally, treatment with either nicotinamide or VPA had no effect on GFP expression of U87 cells transduced with either RGD4C/AAVP-*CMV-GFP* (Figure 2A) or RGD4C/AAVP-*Grp78-GFP* (Figure 2B). These results show that TSA and SAHA, both Zn^2+^ binding inhibitors of HDACs class I and II, restore GFP expression from RGD4C/AAVP-*CMV* in U87 cells; whereas, nicotinamide, a class III HDAC inhibitor, and VPA, an inhibitor of class I HDACs, had no effect on gene expression from the RGD4C/AAVP-*CMV-GFP* phage vector. Based on these observations we can postulate the involvement of HDAC class II in gene expression silencing from RGD4C/AAVP-*CMV-GFP* in U87 cells. Interestingly, induction of RGD4C/AAVP-*Grp78*-guided gene expression by TSA and SAHA treatment was also reproduced in the 9L cells transduced with RGD4C/AAVP-*Grp78-GFP*, and consistently with U87 cells, only MFI was increased with no effect on GFP positive cells (Figure 2C). Again, nicotinamide and VPA inhibitors had no impact (Figure 2C). In contrast, TSA, SAHA, nicotinamide, and VPA treatment of 9L cells transduced with RGD4C/AAVP-*CMV-GFP* had no significant effect on GFP expression (data not shown). Consequently, we investigated an additional mechanism regulating the *CMV* promoter silencing, the promoter methylation status, to determine whether gene expression from the RGD4C/AAVP-*CMV* phage in 9L cells can be rescued by DNA methylation inhibitors. Thus, treatment with various concentrations of the DNA methylation inhibitor 5-Azacytidine (5-Aza) resulted in a dose dependent increase of GFP expression in 9L cells transduced with RGD4C/AAVP-*CMV-GFP* (Figure 3A). Addition of 20 µM of 5-Aza increased the percentage of GFP positive cells to 17.5% compared to 3.5% of control untreated cells, and boosted the MFI by 8.9-fold. In contrast, 5-Aza treatment of 9L cells transduced with the RGD4C/AAVP-*Grp78-GFP* phage resulted in no significant change in GFP expression (Figure 3B). Extensive methylation of the *CMV* promoter sequences has previously been reported [12,13,16,20]. Chromatin structural alterations in the CMV promoter region were also described following suppression of expression of genes stably delivered by AAV vectors carrying the CMV promoter [21,22].

HDACs are upregulated in cancer. Therefore, HDAC inhibitors could be combined to restore RGD4C/AAVP-*CMV* efficacy or enhance RGD4C/AAVP-*Grp78* in cancer cells specifically. Another interesting observation was a dose-dependent induction of the *Grp78* promoter by TSA and SAHA in both 9L and U87 cells at the MFI level only, without any effect on the GFP positive cells. These data show that the GFP positive cells remain stable over time in RGD4C/AAVP-*Grp78-GFP*-transduced cells, confirming the absence of *Grp78* promoter silencing and that treatment with HDAC inhibitors further enhance gene expression from RGD4C/AAVP-*Grp78*. Activation of the *Grp78* promoter by TSA in various cancer cells has been reported [23]. Moreover, *Grp78* induction by HDAC inhibitors was detected in xenograft models and biopsies from breast cancer patients undergoing TSA treatment [23]. HDAC inhibitors are considered potential candidates for cancer treatment [24]. For example, SAHA has recently been approved by the US Food and Drug Administration as a new class of anti-cancer drugs with potential against cutaneous T cell lymphoma [25]. SAHA has also been used in phase I and II clinical trials for hematological malignancies and solid tumors [26,27]. Nevertheless, HDAC inhibitors are considered poor drugs against solid tumors [26,28]. 

**Figure 4:**
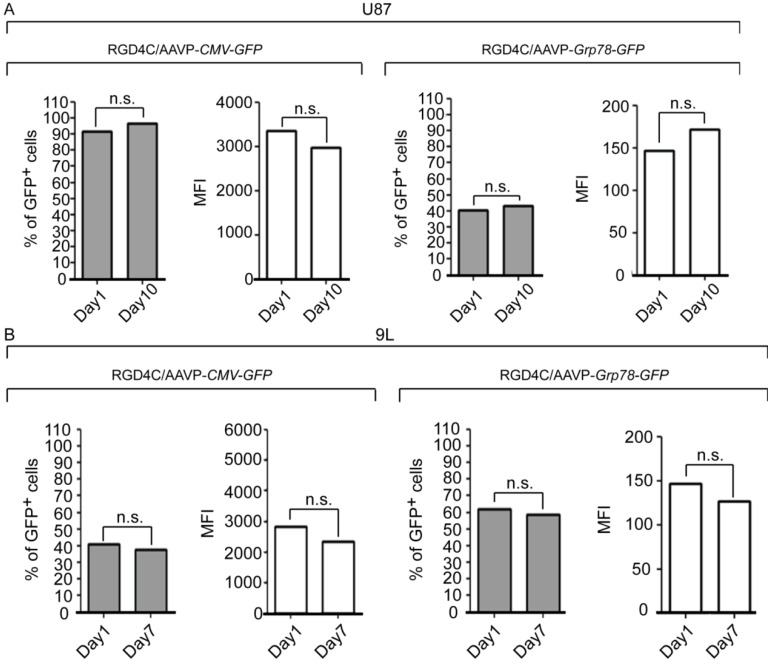
Assessment of the effect of cell confluence on AAVP-driven gene expression in stably transduced cells. U87 and 9L populations of pooled clones stably transduced with RGD4C/AAVP-*CMV-GFP* or RGD4C/AAVP-*Grp78-GFP*, were plated at 300,000 cells/well in 6 well plates and flow cytometry analysis was performed to quantify GFP expression over the indicated times. Shown are percentage of GFP positive cells and MFI derived from transduction with either the RGD4C/AAVP-*CMV-GFP* or RGD4C/AAVP-*Grp78-GFP.* Day one represents 70% confluent cells while days 10 or 7 are the upper-limit time points at which overgrown cells could still be kept alive The experiments were repeated three times, in triplicate. n.s. = non-significant.

*Grp78* is a stress-inducible gene that supports cell survival and drug resistance [29] and cancer cell chemoresistance increases with stress mediated by cell confluence. Therefore, we investigated whether cell confluence induces *Grp78* activity. Transduced U87 and 9L cells were grown until 70% confluence. Subsequently, GFP expression analysis by flow cytometry over time revealed that the percentage of GFP positive cells and MFI remained constant in both U87 and 9L cells transduced with either RGD4C/AAVP-*CMV-GFP* or RGD4C/AAVP-*Grp78-GFP* vectors (Figure 4). These data provide evidence that cell confluence does not affect the RGD4C/AAVP-*Grp78* activity. 

## 3. Experimental

### 3.1. Cell Culture

The human U87 glioblastoma cell line was obtained from the Cancer Research UK and the rat 9L glioblastoma cell line was kindly provided by Dr. Hrvoje Miletic, University of Bergen, Norway. These cell lines were maintained in Dulbecco’s Modified Eagle Medium supplemented with Fetal Bovine Serum (FBS), l-glutamine, penicillin, and streptomycin.

### 3.2. Vector Construction and Phage Production

To generate the double-targeted RGD4C/AAVP-*Grp78-GFP* and targeted RGD4C/AAVP-*CMV-GFP* vectors carrying the *puro^R^* resistance gene, a puromycin resistance cassette was cloned into the *Sac*I site of AAVP vector located next to the AAV transgene cassette. First, the 1,168 bp fragment containing the puromycin resistance gene under the control of *SV40* early enhancer/promoter was released from the pGL4.20 plasmid (promega) by *BamH*I and *Sal*I double restriction digestion then ligated to *Sac*I linkers and subsequently inserted into *Sac*I site of the RGD4C/AAVP-*Grp78-GFP* and RGD4C/AAVP-*CMV-GFP* phage plasmids. Phage viral particles were amplified as described [7] then expressed as bacterial transducing units (TU/μL).

### 3.3. Generation of Stably Transduced Cells

The U87 or 9L cells, 70% confluent, were transduced with either double targeted RGD4C/AAVP-*Grp78-GFP* or targeted RGD4C/AAVP-*CMV-GFP* phage encoding the *GFP* reporter gene and carrying the *puro^R^*, at 2 × 10^6^ TU/cell. At day three, post-vector transduction, cells were trypsinized and suspended in medium containing an appropriate dose of puromycin (1 µg/mL for U87 cells and 7 µg/mL for 9L cells). Parental non-transduced cells were used as controls. The medium was removed and replaced with fresh medium containing puromycin every two to three days. After two weeks, all control cells were killed and puromycin-resistant single cell clones, which were derived from single cells, were monitored under the microscope. When the puromycin-resistant cell clones were visible, they were pooled to produce a population of stably transduced cells. All stable selected cells were maintained in medium containing puromycin, imaged and monitored under a fluorescent microscope every two to three days, then further analyzed by FACS.

### 3.4. Fluorescence Activated Cell Sorting (FACS)

The percentage of cells expressing GFP following stable cell transduction with either RGD4C/AAVP-*Grp78*-*GFP* or RGD4C/AAVP-*CMV-GFP* was determined by using FACS. U87 and 9L cells were seeded in 6 well plates at a density of 3 × 10^5^ cells/well. After 48 h, cells were resuspended in PBS containing 0.5% FBS, then analyzed by using a FACScalibur instrument. A total of 20,000 individual cells were mounted within a manual gate in each condition. 

To validate reactivation of the *CMV* promoter activity, stably transduced U87 and 9L cells as well as parental control cells, were seeded in 6 well plates. After 24 h, cells were treated with DNA methyltransferase or HDACs inhibitor drugs, then monitored by fluorescent microscope. Afterwards, cells were washed, trypsinized and resuspended in PBS containing 0.5% FBS then analyzed by FACS. The mean fluorescence intensity (MFI), used to define the fluorescence intensity of each cell population, and the percentage of GFP positive cells were calculated by normalizing to the control (parental cells grown in similar condition). Data were analyzed using FlowJo software.

### 3.5. Statistical Analyses

Statistical analyses were performed by using GraphPad Prism software (version 5.0). Error bars represent standard error of the mean (s.e.m). *p* values were generated by ANOVA and denoted as follows: * *p* < 0.05, ** *p* < 0.01 and *** *p* < 0.001.

## 4. Conclusions

Our findings show that histone deacetylation and DNA methylation are involved, at least in part, in the long-term gene expression silencing from the parental RGD4C/AAVP bacteriophage gene delivery vector. Specifically, we have presented a novel strategy to advance gene transfer to cancer cells by bacteriophage by combining AAVP with inhibitors of histone deacetylation and DNA methylation. Additionally, our findings prove that combination of *Grp78-*guided gene expression and HDAC inhibitors provides novel and most suitable strategy to enhance cancer gene therapy by the AAVP phage-derived vector, and should be considered for clinical applications against solid tumors. 
